# A Case of Concurrent Behçet Disease and Erythema Multiforme in a Young Adult

**DOI:** 10.7759/cureus.102652

**Published:** 2026-01-30

**Authors:** Yonathan Daniel, Gabrielle Knauer

**Affiliations:** 1 Department of Internal Medicine, Bellevue Hospital Center, New York City, USA; 2 Department of Medicine, Lewis Katz School of Medicine, Temple University, Philadelphia, USA

**Keywords:** behçet disease, comprehensive diagnostic criteria, corticosteroid therapy, discharge planning, erythema multiforme (em), immune-mediated disease, mucocutaneous ulcers, multidisciplinary care team

## Abstract

Behçet disease (BD) and erythema multiforme (EM) are rare, immune-mediated, clinically diagnosed conditions that can present with overlapping mucocutaneous findings and symptoms. We report a diagnostically challenging presentation of concurrent BD and EM characterized by oral ulcers, genital lesions, and targetoid bullous acral lesions in a 23-year-old African American man. This case highlights the risk of premature diagnostic closure and emphasizes that histopathology is supportive rather than determinative in diagnosing either of these conditions. Management required multidisciplinary input and high-dose steroids. We integrated the clinical and histopathological morphology of the patient’s mucocutaneous lesions with the temporal pattern of his symptom progression to confirm a diagnosis of BD with co-occurring EM. This case underscores the importance of integrating criteria-anchored diagnosis with biopsy interpretation and reinforces discharge processes as therapeutic interventions. Notably, the patient was lost to follow-up and returned three months later with similar symptoms, highlighting the importance of discharge planning in preventing relapse.

## Introduction

Behçet disease (BD) is a relapsing, multisystem vasculitis characterized by recurrent oral and genital ulcers, ocular inflammation, and cutaneous manifestations, with potential vascular, neurologic, and articular complications [1]. Sometimes called the Silk Road disease, its prevalence is highest in countries along this ancient trade route (e.g., Türkiye, Iran, Iraq) and is relatively rare in Northern Europe and Africa [1].

Erythema multiforme (EM) is an acute, immune-mediated mucocutaneous syndrome most often triggered by herpes simplex virus (HSV) and characterized by target lesions and, variably, mucosal erosions [2]. Although historically framed as an immune-complex (type III hypersensitivity) phenomenon, contemporary understanding emphasizes a cell-mediated (type IV hypersensitivity) response, particularly in HSV-associated EM [2,3].

Clinical overlap between BD and EM, painful oral ulcers, ocular irritation, and genital involvement, can cause diagnostic confusion, especially because neither condition has a single definitive test. Accurate diagnosis typically rests on pattern recognition over time, supported (but not dictated) by histopathology and the exclusion of mimics [1-3].

We report a case of a young man with simultaneous flares of BD and EM. This case highlights a rare clinical presentation, as simultaneous occurrences of these conditions are infrequently reported. This unique occurrence underscores the following three teaching points: be wary of premature diagnostic closure when mucosal disease predominates, integrate histopathological findings within a broad clinical framework, and align inpatient gains with outpatient maintenance therapy and care-transition infrastructure to reduce relapse.

## Case presentation

A 23-year-old African American man with a remote, episodic history of untreated oral aphthae presented with eight weeks of escalating odynophagia, dysphagia to solids and liquids, and 15-lb weight loss. He also reported two weeks of new acral eruptions of painful, targetoid plaques and bullae, which he had never experienced before. Review of systems was positive for fatigue and negative for fever, joint pain, hematuria, hematochezia, abdominal pain, or ocular symptoms.

For eight years, he had recurrent painful oral and genital ulcers, episodic targetoid rashes, and one prior episode of uveitis, typically treated in emergency settings with short steroid bursts. He had no established rheumatology care and no prior maintenance immunosuppression. He denied taking any medications or supplements, reported no history of sexual activity, used marijuana daily, and used no other substances.

Clinical examination

**Figure 1:**
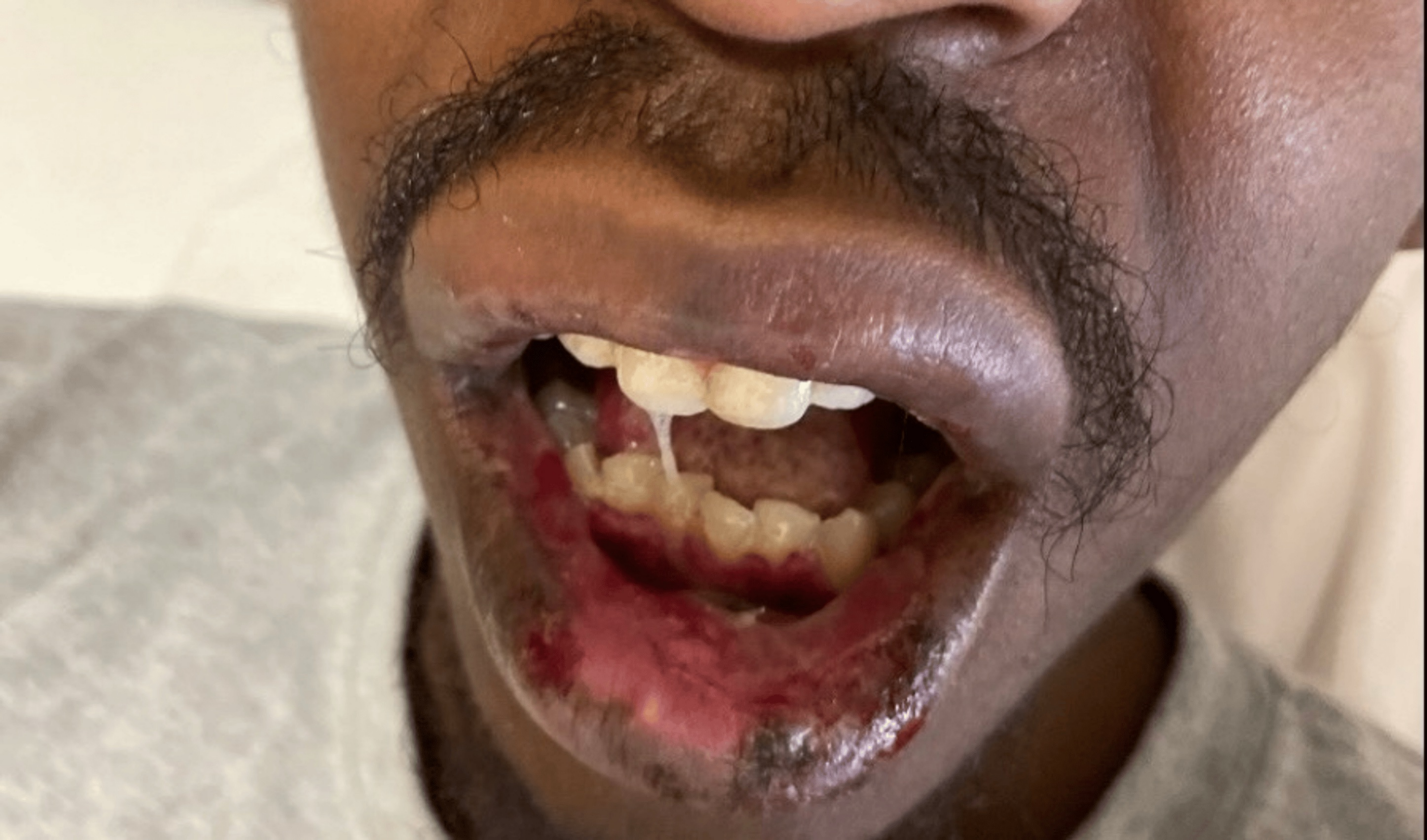
Oral and perioral erosions on admission. Diffuse erosive stomatitis with hemorrhagic crusting of the lips and buccal mucosa.

**Figure 2:**
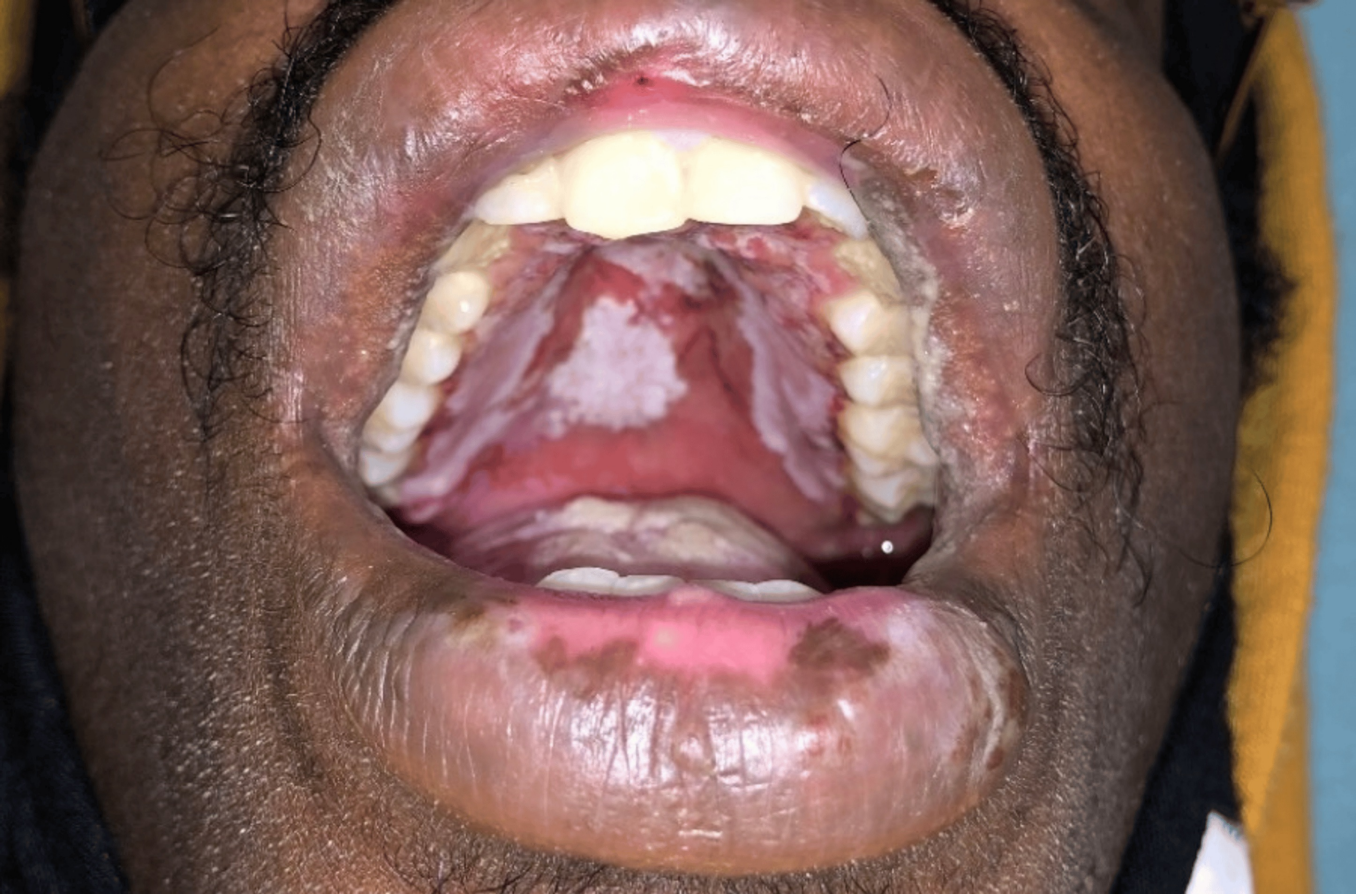
Hard palate ulcerations with pseudomembranous exudate. Extensive erosions and white exudate across the hard palate with surrounding erythema.

**Figure 3:**
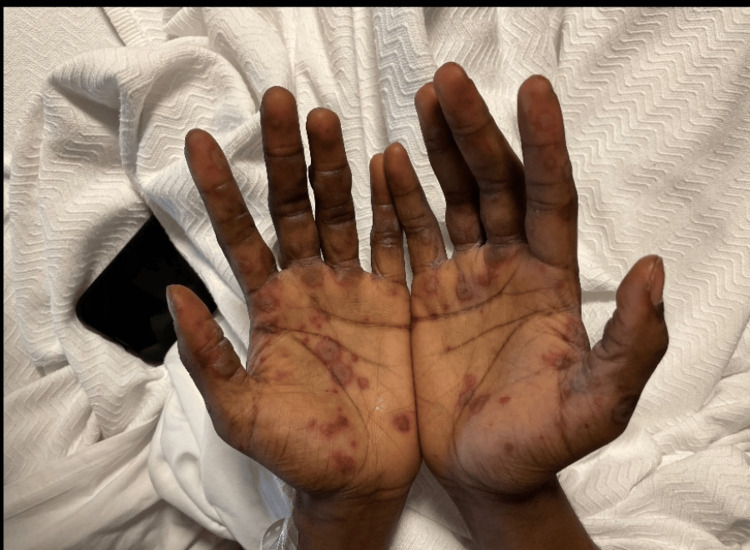
Acral targetoid and bullous lesions of the hands. Symmetric, erythematous to violaceous targetoid plaques and vesiculobullous lesions involving the palms.

**Figure 4:**
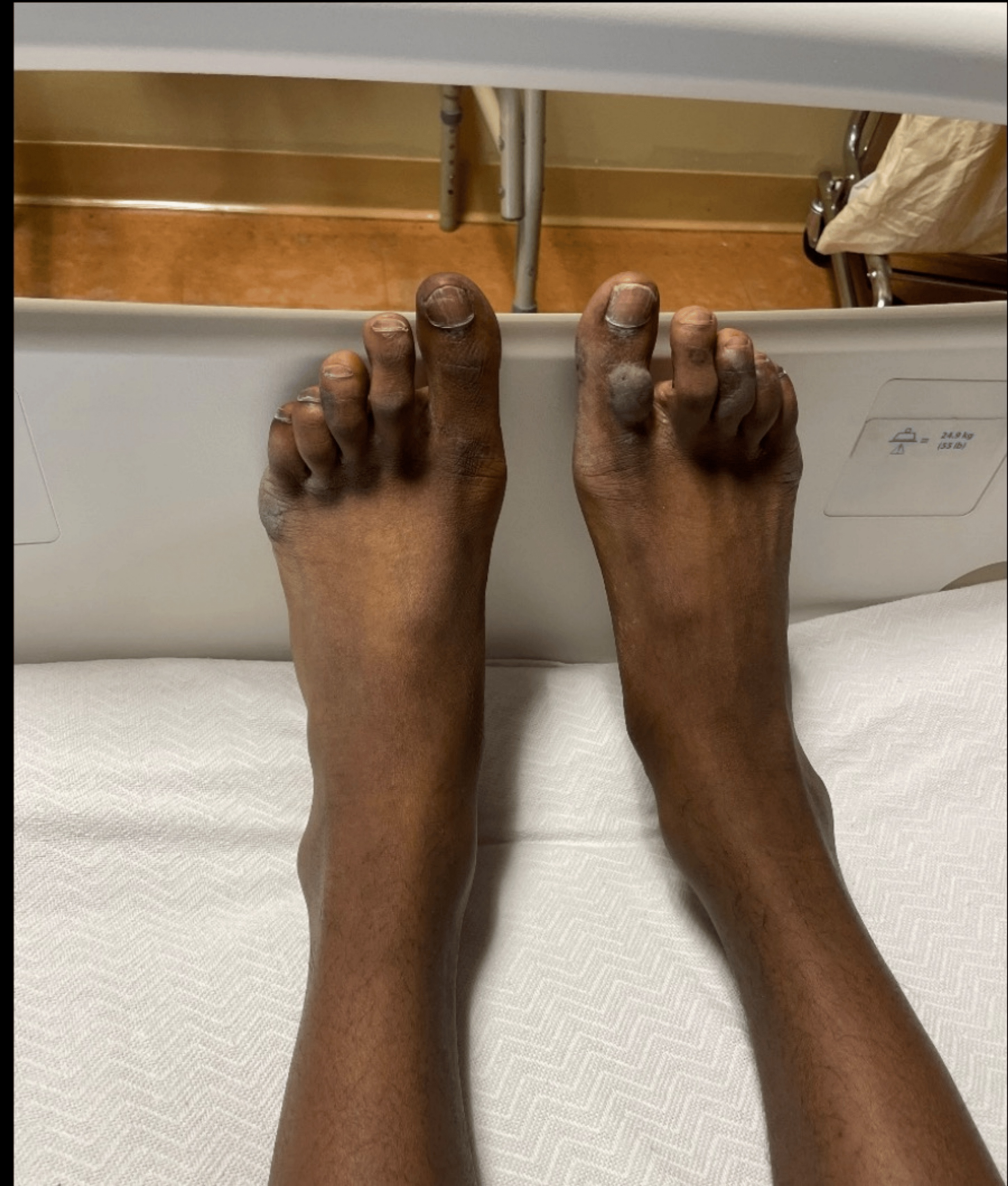
Chronic hyperpigmented acral scarring. Residual hyperpigmented macules and scars on the toes at various stages of healing.

On arrival, he was afebrile and tachycardic, with normal blood pressure and oxygen saturation. Oral examination showed diffuse erosive stomatitis with hemorrhagic crusting of the lips (Figure 1), oral thrush, pseudomembranous exudate of the hard palate, and multiple aphthous-type ulcers on the buccal mucosa, tongue, and hard palate (Figure 2). The skin examination demonstrated symmetric acral targetoid lesions, some bullous, on the hands and forearms; there was no confluent epidermal detachment (Figure 3). Examination of the genitals and lower extremities revealed hyperpigmented scars compatible with prior ulceration (Figure 4). No focal neurologic deficits were present.

Diagnostic workup

Laboratory testing showed systemic inflammation without cytopenias. C-reactive protein (CRP) was 32 mg/L, and erythrocyte sedimentation rate (ESR) was 40 mm/h. Sodium was 133 mmol/L, and the remainder of his labs were unremarkable. Contrast-enhanced computed tomography (CT) of the neck was also unremarkable. Esophagogastroduodenoscopy (EGD) demonstrated diffuse esophagitis with friable ulcers in the upper and mid-esophagus; biopsy was deferred due to risk of hemorrhage. Infectious testing, including herpes simplex virus (HSV) polymerase chain reaction (PCR) from multiple mucocutaneous sites, was negative. Acral skin and oral mucosa biopsies were obtained with the understanding that pathology reports would require several days.

Therapeutic interventions and hospital course

Initial therapy targeted a broad differential as follows: oral valacyclovir, nystatin, a proton-pump inhibitor (PPI), multivitamin supplementation, acetaminophen, and morphine as needed. With worsening dysphagia and poor oral intake on hospital day two, medications were converted to IV formulations while the team worked to rule out competing diagnoses. Stevens-Johnson syndrome and fixed drug eruption were considered unlikely given discordant lesion morphology, limited body-surface area, and absence of culprit medications. Pemphigus vulgaris and bullous pemphigoid were also unlikely based on examination findings and lesion distribution. Infectious mucositis was less likely given the negative infectious workup and HSV testing; thrush was present but insufficient to explain the acral targetoid eruption. Inflammatory bowel disease-related aphthae and reactive arthritis were considered less likely given the absence of gastrointestinal, articular, or enthesitis features.

After a multidisciplinary review by dermatology, rheumatology, gastroenterology, otolaryngology, and retrieval of outside medical records consistent with Behçet disease, the patient was treated with high-dose methylprednisolone for a presumed Behçet disease flare on hospital day three. His symptoms improved markedly over 48 h, and he was discharged on hospital day five. Histopathology reports on the biopsied tissues were reported after discharge, revealing papillary dermal edema in the skin and dense neutrophilic perivascular infiltrate with vasculitic changes in the oral mucosa. Direct immunofluorescence (DIF) was negative for intercellular or basement-membrane deposition. With competing diagnoses excluded and rapid improvement on steroids, a final working diagnosis was agreed upon after discharge by medicine, dermatology, and rheumatology as follows: an acute-on-chronic Behçet disease flare complicated by co-occurring erythema multiforme. Discharge planning included expedited follow-up with rheumatology, dermatology, and ophthalmology to initiate maintenance therapy for Behçet disease, along with nutrition follow-up and counseling regarding symptom surveillance. Despite outreach from the care-transitions team, the patient did not attend outpatient visits and was readmitted three months later with similar symptoms.

## Discussion

Why this case matters

First, it demonstrates true coexistence of Behçet disease and erythema multiforme rather than mislabeling one as the other. In Behçet disease, the most commonly used diagnostic tool is the International Study Group (ISG) criteria, which emphasize recurrent oral ulcers plus two or more of genital ulcers, ocular inflammation, or characteristic skin lesions [1]. Our patient met criteria through recurrent oral and genital aphthae and a documented prior episode of uveitis. The biopsy findings were also supportive of Behçet disease, given the clinical context. The diagnosis of erythema multiforme was based on the presence of round, well-defined, concentric target lesions in a symmetric, acral-predominant distribution with limited epidermal detachment. Histopathology showing interface dermatitis was nonspecific but consistent with erythema multiforme in the appropriate clinical setting [2,3].

Second, this case illustrates that biopsy findings are supportive, not determinative. In both conditions, the associated histopathologic findings serve an ancillary role to the clinical exam and history. Clinicians are best served by integrating histopathology with evolving morphology and diagnostic criteria rather than over-relying on biopsies when managing immune-mediated mucocutaneous disease [4-6]. Third, this case reframes discharge as part of treatment in chronic immune-mediated disorders - reliable pathways for access, affordability, and accountability are as crucial as the steroid-sparing agent itself.

Applying diagnostic criteria

Behçet disease and erythema multiforme are primarily clinical diagnoses because their histopathologic features are variable and often overlap with other conditions. The “classic” finding in erythema multiforme-interface dermatitis with necrotic keratinocytes may be missed if the biopsy is obtained too early or too late in lesion evolution [2,3]. No single stain or tissue feature is pathognomonic for erythema multiforme, and similar histologic patterns occur in viral exanthems, lupus erythematosus, and early Stevens-Johnson syndrome.

Similarly, the typical findings in Behçet disease of small-vessel vasculitis, perivascular inflammation, and thrombophlebitis overlap with infectious, drug-induced, and other autoimmune vasculitides [4-6]. Moreover, Behçet disease produces multiple lesion types - papulopustular lesions, erythema-nodosum-like nodules, superficial thrombophlebitis, and oral ulcers - each yielding distinct histologic patterns [4]. Oral ulcers, in particular, are nonspecific because the ulcer base largely consists of granulation tissue. 

Management challenges

The disease course was complicated by fragmented healthcare access. Reliance on episodic urgent-care visits led to repeated corticosteroid bursts without initiation of maintenance immunosuppression, which is the cornerstone of Behçet disease management in patients with systemic involvement [7,8]. According to the 2018 European Alliance of Associations for Rheumatology (EULAR) recommendations, agents such as colchicine, azathioprine, apremilast, or tumor necrosis factor inhibitors should be considered for recurrent mucocutaneous and ocular Behçet disease [7]. Early initiation of these therapies may help prevent recurrence and hospitalization [8].

In contrast, management of erythema multiforme focuses on identifying and eliminating triggers, most often herpes simplex virus, and on symptomatic relief [2,3]. The absence of herpes infection in this patient suggested a noninfectious erythema multiforme flare, raising the possibility of shared immune dysregulation. Whether Behçet disease predisposed him to erythema multiforme-like immune activation remains speculative but highlights the complexity of overlapping immune-mediated syndromes.

Systems of care and preventing readmissions

This case underscores how fragmented care perpetuates morbidity. The patient lacked continuous rheumatology oversight, leading to delayed recognition of his Behçet disease and failure to initiate disease-modifying therapy [5,8]. Additionally, his post-discharge planning was insufficient, consisting primarily of instructions to schedule follow-ups rather than active care coordination. He was ultimately readmitted with preventable disease recurrence, illustrating the importance of structured transitions of care, patient education, and system-level interventions for chronic immune-mediated conditions.

Comprehensive discharge planning should incorporate several key actions. Before discharge, rheumatology and dermatology appointments should be booked and transportation confirmed. Warm handoffs between inpatient and outpatient clinicians are essential; the inpatient team should communicate directly by phone and provide a concise one-page summary outlining diagnostic reasoning and the proposed maintenance plan. Medication bridging should include a steroid taper with explicit parameters for dose escalation if symptoms recur, along with consideration of early colchicine initiation for mucocutaneous Behçet disease [7,8]. Patients should receive clear education on early warning signs, ocular pain or redness, new genital or disabling oral ulcers, and targetoid eruptions, as well as hydration and nutrition strategies and guidance on when to contact the care team.

Accountability for follow-up should be reinforced through care navigation support, social work involvement to address insurance or transportation barriers, and a nurse phone call within 72 h of discharge. Finally, documentation should clearly state that this case represents coexisting Behçet disease and erythema multiforme, ensuring that future clinicians do not stop at the first positive biopsy result when evaluating potential recurrence.

## Conclusions

This case highlights a rare dual presentation of Behçet disease and erythema multiforme in a young adult with severe mucosal disease, underscoring the diagnostic complexity of overlapping immune-mediated conditions. The coexistence of these entities challenges clinicians to maintain a broad differential diagnosis and to integrate evolving clinical, histopathologic, and historical data rather than relying on any single feature in isolation. Biopsy findings should inform, but never dictate, the final diagnosis, as histologic overlap and sampling variability are common in mucocutaneous inflammatory disorders.

Equally important, this case demonstrates that successful inpatient management is only the first step in achieving durable disease control. Sustained remission depends on the timely initiation of maintenance therapy tailored to disease severity, patient adherence, and psychosocial context. Coordinated, multidisciplinary follow-up, including rheumatology, dermatology, ophthalmology, and primary care, is essential to ensure continuity of care, prevent relapse, and mitigate corticosteroid dependence. Finally, robust transitions of care, characterized by proactive scheduling, patient education, and clear communication between inpatient and outpatient teams, are critical to translating short-term inpatient improvement into long-term outpatient stability and improved quality of life for patients with chronic, relapsing immune-mediated disease.
